# Knowledge, Attitudes, and Perceptions of Dental Professionals Regarding Robotics and Artificial Intelligence in Dentistry: A Cross-Sectional Study in Lucknow, India

**DOI:** 10.7759/cureus.78882

**Published:** 2025-02-11

**Authors:** Pratisha Mishra, Arunendra Vikram Singh, Pratyush Kar, Aparna Chaturvedi

**Affiliations:** 1 Public Health Dentistry, Sardar Patel Post Graduate Institute of Dental and Medical Sciences, Lucknow, IND; 2 Anesthesiology, Sanjay Gandhi Postgraduate Institute of Medical Sciences, Lucknow, IND

**Keywords:** artificial intelligence, dentistry, education, robotics, technology

## Abstract

Introduction

Artificial intelligence (AI) and robotics have now come to redefine the face of different healthcare paradigms, including dentistry. AI refers to the development of computer systems that are intended to simulate human intelligence. Robotics applies these systems to achieve tasks. There has been global advancement in AI and robotics; however, their use in Indian health care is still scant. This article evaluates the knowledge, attitudes, and perceptions of dental professionals concerning AI and robotics in the oral health of Lucknow city in India.

Objectives

This study aims to assess the awareness of dental professionals regarding AI and robotics, as well as evaluate their attitudes toward integrating AI into dental practice. Additionally, it seeks to identify gaps in knowledge related to AI applications in dentistry and explore potential barriers to its adoption in clinical practice.

Methods

This cross-sectional, observational survey included 285 dental professionals (independent practitioners, postgraduate students, and faculty members) in Lucknow, India. A pre-validated questionnaire with 18 closed-ended questions was used for data collection via Google Forms (Google, Inc., Mountain View, CA, USA). The sampling method was non-probability snowball sampling, and data analysis was conducted using the Statistical Package for the Social Sciences (IBM SPSS Statistics for Windows, IBM Corp., Version 26.0, Armonk, NY). Chi-square tests were used to assess associations between demographics and responses. A p-value <0.05 was considered statistically significant. Validation metrics for the questionnaire were included to enhance credibility.

Results

Most participants had knowledge of robotics (88.8%) and AI (96.5%). However, 14.4% to 20.4% of respondents lacked awareness of specific AI applications. A majority (77.5%) agreed that AI facilitates computer-aided design and manufacturing (CAD/CAM) processes, yet 73% expressed skepticism about AI fully replacing dentists.

Conclusion

Dental professionals in Lucknow generally support AI and robotics integration but exhibit significant knowledge gaps. Structured AI education and continuous professional training are essential for successful integration into dental practice. Ethical considerations, including data privacy, algorithmic bias, and AI accountability, should be addressed in future policies.

## Introduction

The human brain has an inimitable architecture of nets of interconnected neurons that enable it to convey information throughout the body - a function that has captivated scholars and scientists since the very dawn of time. Through the years, tremendous technological inventions have been made to develop the functions of human intelligence, which resulted in the birth of artificial intelligence (AI). It is generally defined as a field of science and engineering concerned with the computational understanding of what has commonly been called intelligent behavior and with the creation of artifacts that exhibit such behavior [1]. Machine learning (ML) is a branch of AI that applies computational algorithms for analyzing data sets with minimal explicit programming to make predictions or decisions. Deep learning (DL) is an area of ML that has recently become quite popular, mainly because large datasets are more readily available, high-performance computational resources exist, and open-source software frameworks exist [2].

The quick pace of development has made AI and robotics play such an important role in everyday activities. Robotics refers to the idea, design, construction, use, or application of robots. In this regard, it collaborates well with AI - the concept that creates computer programs that resemble human intelligence and can be used for such purposes as voice recognition, decision-making, and disease diagnosis [3]. AI has advanced considerably and has been utilized in many areas, including industry and finance, health, and meteorology [4].

India underuses its full potential in AI and robotics despite technological advances. Indeed, now 87% of India's 1.3 billion people have access to the internet, while mobile phone use is reported by 70% of those aged 18 to 60. However, an overwhelming proportion of doctors and dentists still do not know the concepts, capabilities, and personal and professional impacts related to AI [5].

AI use in medicine has increased, while its application in dental practice requires caution. Robotics is used in various dental surgical procedures, while there is scope for robotics in diagnostic, preventive, and restorative methods [6]. The use of modern digital technologies in preventive dentistry and oral healthcare is exponentially increasing worldwide and is in vogue.

Tomorrow's health providers and professionals will face the challenges of the AI revolution, which requires an adequate understanding of the current technologies and developments in the near future. The number of research articles on AI in radiography is increasing, but evidence-based recommendations and position statements are still pending for this field in dentistry [7]. Harmful perceptions of poor career choices due to inaccurate or a lack of information regarding AI in future clinical practice may arise.

Most of the AI studies in dentistry present small sample sizes, which indicates that clinical acceptance is still a challenge for many applications. There is a need to give proper, objective, and updated information to students as well as clinicians in this transitional period [8]. The understanding of AI's role in dentistry in the future can only be gauged by assessing the current attitudes of clinical dentists and undergraduate dental students toward AI. This would improve the integration of robotics and AI into dental care procedures by educating students, graduates, and professionals about the wide range of skills necessary to handle complex digital dentistry equipment [9].

Ethical and professional barriers to AI adoption

India's healthcare system underutilizes AI and robotics despite its digital advancements. A significant proportion of healthcare professionals remain unaware of AI’s capabilities. While AI applications have expanded in medicine, their role in dental practice requires structured educational frameworks. Concerns include privacy, reliability, and accessibility of AI-integrated systems.

The purpose of the present cross-sectional study was to evaluate the knowledge, attitude, and perception (KAP) of dental professionals in Lucknow city regarding the role of robotics and AI in the delivery of oral healthcare services. The latest technologies are being used nowadays and aim to contribute to the growing pool of literature on AI in dentistry, providing insights into how these technologies can be assimilated in a better way into clinical practice.

## Materials and methods

This observational, cross-sectional study was conducted to assess the KAP of dental professionals in Lucknow city in India regarding the role of robotics and AI in oral healthcare. A total of 285 research associates, including independent dental practitioners, postgraduate students, and faculty members from dental colleges in Lucknow city, participated in the study conducted over a period of six months. The techniques used here were the non-probability sampling of the snowball sampling technique, which presents a potential selection bias that limits generalizability.

Sampling method and sample size estimation

Using G power (version 3.1.9.6; Heinrich-Heine-Universität Düsseldorf, Düsseldorf, Germany), based on the 95% confidence interval and 5% type I error and response distribution of 56.9%, the sample size was estimated to be 273 and, taking the 5% attrition rate into account, the sample size was increased to 285.

Ethical approval

This research work was approved by the Institutional Ethical Committee, Sardar Patel Postgraduate Institute of Dental and Medical Sciences, Lucknow (PG/212291/IEC/SPPGIDMS). Participants agreed to take part on their own will. Written informed consent was obtained from all participants.

Inclusion criteria

The study population included independent dental practitioners, postgraduate dental students, and faculty members of dental colleges working in Lucknow city.

Exclusion criteria

Participants who did not respond to the communication emails or submitted incomplete responses were excluded.

Data collection tool

A pre-validated, specially designed questionnaire having 18 closed-ended questions was used as the data collection tool. The questionnaire had a structured form. The first section included questions about the respondents, like their gender, education, and years of practice. The second section dealt only with the KAP of the respondents toward robotics and AI, structured into three categories: knowledge, perception, and attitude, respectively, relating to questions 1-6, 7-12, and 13-18. The respondents were asked to answer the questions with "yes," "no," or "I don't know" [10]. For the questionnaire, Google Forms (Google, Inc., Mountain View, CA, USA) was used to send it online through communication channels such as WhatsApp and email. Reminders were sent at regular intervals of two times a week to maximize the number of responses [10].

Statistical analysis

The data was acquired and entered into an MS Excel spreadsheet (Microsoft® Corp., Redmond, WA, USA) for initial organization. Statistical analysis was then conducted using the Statistical Package for the Social Sciences (IBM SPSS Statistics for Windows, IBM Corp., Version 26.0, Armonk, NY). Descriptive statistics along with appropriate significance tests were employed to assess the data. A p-value ≤ 0.05 was considered statistically significant, while p-values < 0.01 were considered highly significant. The study maintained an 80% power and a 95% confidence level. For the KAP analysis, a chi-square non-parametric test was employed to assess relationships between key variables such as years of experience, gender, and professional roles among participants.

## Results

Distribution of study subjects by demographic characteristics

Gender Distribution

**Figure 1:**
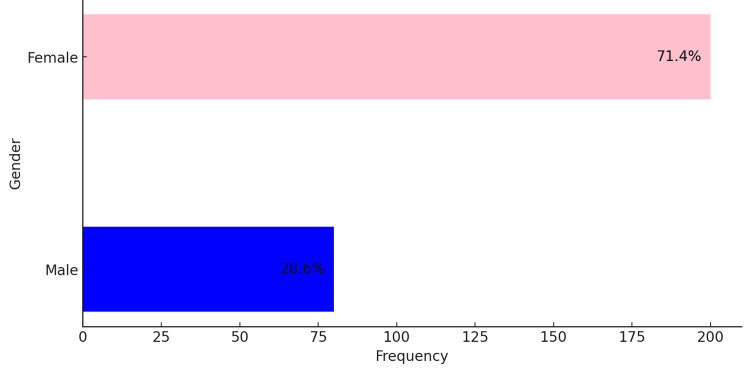
Distribution of study subjects based on gender

This distribution highlights a predominance of female participants. Out of 285 participants, 207 were female, accounting for 71.4% of the study population, while 78 were male, representing 28.6%. This distribution indicates a significant female dominance among the study participants (Figure 1).

Years of Practice

**Figure 2:**
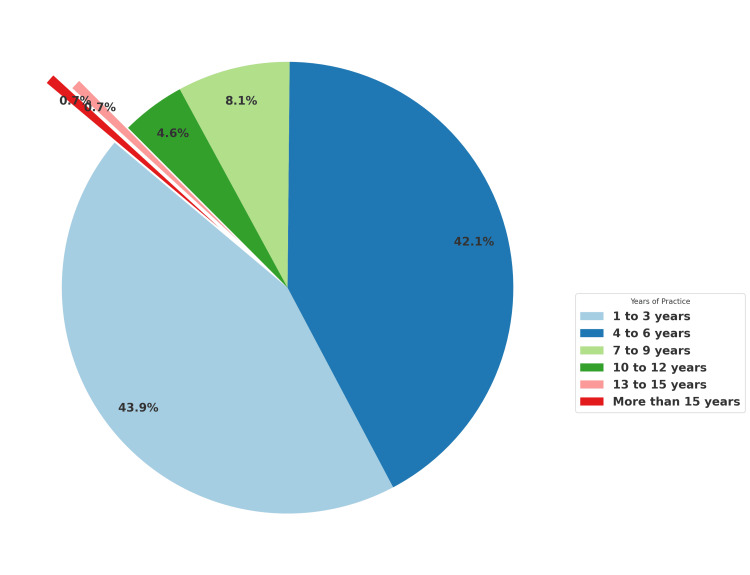
Distribution of study subjects based on years of practice

The distribution of study subjects based on years of practice demonstrated that a majority of participants had relatively fewer years of experience in the field. Specifically, 125 participants (43.9%) had one to three years of practice, and 120 participants (42.1%) had four to six years of experience. Those with seven to nine years of experience comprised 8.1% of the sample (23 participants), and 13 participants (4.6%) had 10 to 12 years of experience. The smallest groups were those with 13 to 15 years and more than 15 years of experience, each representing only 0.7% of the study population (two participants in each group). These findings suggest that the study primarily involved younger professionals or those relatively new to the field (Figure 2).

KAP towards robotics and AI

Knowledge

The knowledge-related questions revealed that most participants were aware of robotics and AI in dentistry. Specifically, 253 (88.8%) of participants were aware of robotics, and 275 (96.5%) were aware of AI. However, when asked about specific applications, such as the use of robots in measuring vital signs and AI in interpreting radiographs, 41 (14.4%) to 58 (20.4%) of participants indicated they were unaware. The chi-square test confirmed that the awareness levels for most questions were statistically significant (p < 0.01), indicating a high level of general knowledge among participants (Table 1).

**Table 1:** Knowledge, attitude, and perception towards robot technology and artificial intelligence * means statistically significant at p-value <0.05; ** means statistically significant at p-value <0.01; and *** means statistically significant at p-value ≤0.001. CBCT: cone-beam computed tomography; MRI: magnetic resonance imaging; CAD/CAM: computer-aided design/computer-aided manufacturing

Questions	Don’t Know n (%)	Yes n (%)	No n (%)	Chi-square	p-value
Q1 Are you aware about robotics?	5 (1.8)	253 (88.8)	27 (9.5)	102.34	0.001^***^
Q2 Are you aware about artificial intelligence?	2 (0.7)	275 (96.5)	8 (2.8)	113.44	0.001^***^
Q3 Robot technology is used to assist with patient diagnosis and the development of an integrated treatment plan.	41 (14.4)	201 (70.5)	43 (15.1)	94.20	0.001^***^
Q4 Robots are used in measurement of vital signs such as pulse, breathing, temperature, blood pressure, and ECG.	50 (17.5)	194 (68.1)	41 (14.4)	44.24	0.001^***^
Q5 Artificial intelligence is used for examinations and their interpretation, e.g., radiographs, CBCT, MRI, differentiation, between vital and pathological signs.	38 (13.3)	207 (72.6)	40 (14)	92.55	0.001^***^
Q6 Artificial intelligence is used in pathology, accurate reading of tissue samples, diagnosis.	58 (20.4)	185 (64.9)	42 (14.7)	31.69	0.001^***^
Q7 Artificial intelligence is used in detection of oral cancer in its early stages, such as during health campaigns.	79 (27.7)	164 (57.5)	42 (14.7)	0.873	0.321
Q8 Robotics/AI use in oral health and preventive dentistry is beneficial.	62 (21.8)	189 (66.3)	34 (11.9)	18.39	0.001^***^
Q9 Automated surgical robot in the area of maxillofacial surgery is that which supports the surgeon in performing a certain operation or may act as a surgeon’s assistant.	43 (15.1)	190 (66.7)	52 (18.2)	19.47	0.001^***^
Q10 In the field of orthodontics, artificial intelligence can provide a more accurate digital view of the mouth than the traditional method and predict the movement of teeth and the final treatment of teeth work applications with wire, as opposed to the laboratory.	37 (13)	214 (75.1)	34 (11.9)	97.98	0.001^***^
Q11 In endodontic treatment, working robots may reduce possible treatment errors and increase the quality of treatment.	40 (14)	181 (62.5)	64 (22.5)	12.90	0.027^*^
Q12 Robotics/AI may contribute to predicting the correct place in cases of dental implants through a 3d view before and during the process through an integrated simulation system.	40 (14)	199 (69.8)	46 (16.1)	53.32	0.001^***^
Q13 Robotics/AI facilitates CAD/CAM and process of fabricating complete denture.	38 (13.3)	221 (77.5)	26 (9.1)	74.27	0.001^***^
Q14 Can AI replace the dentist permanently?	24 (8.4)	53 (18.6)	208 (73)	79.22	0.001^***^
Q15 AI facilitates the storage of patient information, data and accessibility to it, quickly and accurately.	27 (9.5)	230 (80.7)	28 (9.8)	99.23	0.001^***^
Q16 Can robots contribute to increased career productivity, medical education and awareness in the community and individuals?	34 (11.9)	148 (51.9)	103 (36.1)	82.92	0.001^***^
Q17 Would you recommend treatment with robotics/AI?	60 (21.1)	114 (40)	111 (38.9)	0.43	0.631
Q18 Would you prefer treatment done with robotics/AI on yourself, if needed?	52 (18.2)	95 (33.3)	138 (48.4)	8.341	0.003^**^

Attitude

Participants generally exhibited a positive attitude towards the integration of robotics and AI into dental practice. For example, 221 (77.5%) of participants agreed that robotics/AI facilitates the computer-aided design and manufacturing (CAD/CAM) process and the fabrication of complete dentures. However, skepticism was evident regarding the potential for AI to fully replace dentists; 208 (73% of participants) disagreed with the notion that AI could permanently replace human dentists. The chi-square analysis showed statistically significant positive attitudes towards several AI applications (p < 0.01), though there were areas of uncertainty, particularly regarding AI’s role in complex clinical decisions (Table 1).

Perception

In terms of perception, the study found that while there was a positive inclination toward the adoption of robotics/AI, actual confidence in their widespread use was limited. For instance, while 181 (62.5%) of participants believed that working robots could reduce errors in endodontic treatment, 111 (38.5%) were hesitant to recommend robotics/AI in clinical settings. Furthermore, 138 (48.4%) of participants expressed reluctance to undergo treatment with robotics/AI themselves. These findings reflect a cautious approach to the integration of these technologies, with significant concerns about the efficacy and reliability of robotics/AI in clinical environments.

Overall, the study reveals strong awareness and generally positive attitudes toward robotics/AI among dental professionals, though significant reservations remain, particularly regarding the complete replacement of human expertise in dental practice (Table 1).

The results of the study are very evident that knowledge gaps remain, particularly in specific AI applications in diagnosis and treatment planning. Also, it gives an idea about skepticism persisting, despite general acceptance of AI’s role in dentistry.

## Discussion

Recent years have shown developments in technology that closely mimic the functions of the human mind. Robotics and AI are listed among some of the most remarkable achievements in recent times. Several methodologies in AI with a focus on advanced algorithmic models show great promise for applications in the biomedical sciences. For example, it revolutionized the medical field and, nowadays, is applied to diagnosis, radiology, and all procedures used in treatment. It is the same with the discipline of dentistry; none can be excluded since more and more findings are published within the framework of research that involves these technologies [4,10].

AI obviously changed the course of dentistry with digital technologies. The newest AI paradigms include computer vision, DL, cognitive computing, ML, and natural language processing, which could play a critical role in the enhancement of oral healthcare services. These technologies help interpret complex radiographic scans, thereby improving diagnostic precision and minimizing errors, which may lead to more comprehensive detection of craniofacial conditions. Further, Sur et al. demonstrated that 68% of dentists were knowledgeable about ideas concerning AI, 69% believed that AI could be used for diagnosis and treatment planning, and 63% believed that AI would be bright in the near future in India [6]. In the present study, it is observed that approximately 253 (88%) dental professionals were aware of the term robotics, and 275 (96%) were aware of AI, whereas eight (2.8%) dentists did not have any knowledge about robotics.

A few percent of respondents did not know, five (1.8%) for robotics and two (0.7%) for AI. A knowledge gap, as exemplified by questions Q1 and Q2 in Table 1, respectively, may have resulted from limited BDS undergraduate curriculum exposure to robotics and AI technologies, a point well illustrated by Abouzeid et al. With respect to diagnosis and treatment planning (Q3), 43 (15%) had no knowledge, which was consistent with the same study findings [3].

AI has been very promising in the identification and classification of abnormal mucosa, which may be experiencing premalignant or malignant changes, and in diagnosing and treating disorders that could affect the oral cavity. It can detect minute changes at the single-pixel level that might not be visible to the naked eye. Furthermore, AI can precisely compute the genetic predisposition of a significant population for oral cancer. According to a systematic review by Ahmed et al., the benefits of AI in dental diagnostics that would eliminate human errors and enable dental practitioners to observe anomalies with precision were reported. In the meantime, the assistance of a computer-based neural network will reduce dental treatment errors and promote well-informed decisions [4]. More than 50% of the respondents gave affirmative answers to questions Q5, Q6, and Q7 regarding dental abnormality diagnosis, as supported by the evidence found in previous reviews [4].

AI technology has also transformed orthodontic diagnosis, planning, and monitoring treatment [11]. Radiographs and images taken using intraoral scanners and cameras are analyzed with AI for enhanced diagnosis and treatment, with outcomes being more accurate than classical approaches. It further helps in oral maxillofacial surgery, where procedures assisted by AI have been found to be shorter, more precise, and safer with manipulations. Imaging guidance allows for surgical resections to be more accurate than before and so reduces the number of revision procedures. These developments suggest that AI and robotics have a long way to go in dental and surgical practices [10,12]. The findings of this study show that the majority of dental professionals from various age groups held positive perceptions of robotics and AI, as reflected in their responses to questions Q9, Q10, Q11, Q12, and Q13, which pertain to the use of robotics and AI in orthodontics, prosthodontics, surgery, and endodontics.

Several studies and reviews have been conducted on the accuracy, safety, and reliability of microendodontic robots to evaluate them in root canal treatments. The positive overall perception of using microendodontic robots among dental graduates in this study shows promise for incorporating robotics and AI into endodontic practice in the future [13].

As Mupparapu et al. mentioned, AI technologies offer an additional sense of vision to dentists in nanoseconds, thereby improving diagnosis and aiding in the attainment of better results for patients [6,14]. This technology allows for the very accurate printing in 3D of aligners with a treatment plan as a focus to optimize their use in tooth movement through complex algorithms. The integration of digital technologies into Miniscrew-Assisted Rapid Palatal Expander (MARPE) appliances and treatments has led to considerable procedural benefits in terms of precision, customized service, and workflow efficiency [15]. A case report by Viet et al. states that digital tools, including smile design, 3D-printed guidelines, and 3D simulation, helped the patient with a severe gummy smile obtain orthodontic and gingivoplasty treatment. Significant and satisfactory effects were achieved with the procedure without demanding major surgery [16]. The prospects are almost next to nil that AI may eventually replace healthcare professionals and take their place. This is well supported by the conclusions drawn in the current survey for Q14, where the majority of participants showed disagreement that AI can replace dentists in the future.

This study possesses the following strengths, which render it more worthwhile. The study is the first regional study assessing AI perceptions in dental professionals. This is one of the few works focusing on dental professionals' KAP related to dentistry with regard to robotics and AI. It conducted a comprehensive questionnaire that exhaustively covered the whole scope of relevant issues, which led to a holistic examination by the participants with regard to their understanding and perception. A chi-square non-parametric test was proven to be helpful in robust statistical analysis to verify associations across the main variables, such as years of experience, gender, and professional roles, and how participants responded. In a nutshell, these strengths establish the importance of the research findings and compel further studies to hold a deep assessment on a larger scale and in a variety of settings as regards the integration of robotics and AI in dental practice.

Despite the promising findings, this study has a couple of limitations that need consideration. First, in terms of sample size, as the sample size is very small, it may limit generalizability. Although the study provides worthwhile insights into the nature of KAP of dental professionals in Lucknow, the findings may not fully represent the broader population of dental professionals elsewhere in other regions or countries. Another limitation is that it's self-reported data, always with the risk of response bias: people overestimate their knowledge or provide socially desirable answers, thus influencing the outcome. Also, since it is a cross-sectional study, it just captures the cross-section of participants' perceptions and knowledge at one point in time and does not allow for any sort of assessment regarding changes or trends over time. The study found that most of the participants had less than six years of experience, and it may cause experience bias.

Generalization of research is also fundamental. There is a need to study a large and diverse population in establishing the generalizability, reliability, and accuracy of AI technologies in dentistry. Such investigations would also seek to evaluate the long-term effects of integrating AI in healthcare on clinical outcomes and efficiency of practice. Besides the above issues, the ethical implications of AI and the ethical challenges of AI adoption (data privacy, liability, and bias in AI models) were not directly addressed in this study. Future research should examine these barriers, particularly in data management, necessitating comprehensive policies and regulations. It is then upon seeing the synergy of research and policy-making in efforts to give contours to guidelines for the protection of patient data and responsible and transparent use of AI.

## Conclusions

The detection of head and neck abnormalities can change significantly with the advent of robotics and AI, especially in providing interpretations for very complex radiographic scans and improving the accuracy of diagnoses. However, people have different views about what AI's future might hold in the health sector. Many, especially the stakeholders, feel that AI brings with it many opportunities for medicine and dentistry, while others question whether it is reliable enough to replace healthcare providers entirely. The outcomes of this study conclude that dental specialists have a sound perception of AI concepts and understand the role of AI in improving diagnostic precision, treatment efficiency, and overall outcomes in dental practice. Though the majority of graduates in dentistry are aware of this advanced technology, there is still some unwillingness to have robotics and AI practiced completely in the clinics. It may be due to a lack of general knowledge of the subject and a lack of training in the same. To bridge this knowledge gap, it is high time that robotics and AI inclusion is made in the dental undergraduate curriculum along with being supported by continuing dental education (CDE) programs through professional experts who are trained in the field and allied to the dental colleges.

A few key areas for future endeavors should be targeted toward ensuring that AI is properly and ethically applied in dentistry. There should be training in robotics and AI technologies in dental education programs with a strong emphasis on practical applications, ethical considerations, and results that might determine clinical outcomes. This would be useful for orienting future dental professionals towards easily and effectively applying AI within their practice. These courses should, therefore, be based on practical and case-based exposure for effective application of these technologies in practice. The governing of the use of AI in dentistry will be important for policy development and implementation. Together, government agencies, dental associations, and other participating stakeholders will need to develop policies that maximize the fruits of AI while keeping patient rights and ethics on top of the agenda.
